# Recent Developments in Synthesis, Properties, Applications and Recycling of Bio-Based Elastomers

**DOI:** 10.3390/molecules29020387

**Published:** 2024-01-12

**Authors:** Manuel Burelo, Araceli Martínez, Josué David Hernández-Varela, Thomas Stringer, Monserrat Ramírez-Melgarejo, Alice Y. Yau, Gabriel Luna-Bárcenas, Cecilia D. Treviño-Quintanilla

**Affiliations:** 1 Institute of Advanced Materials for Sustainable Manufacturing, Tecnologico de Monterrey, Queretaro 76130, Mexico; gabriel.luna@tec.mx; 2 Escuela Nacional de Estudios Superiores, Unidad Morelia, Universidad Nacional Autónoma de México, Antigua Carretera a Pátzcuaro No. 8701, Col. Ex. Hacienda de San José de la Huerta, Morelia 58190, Michoacán, Mexico; aracelimp@enesmorelia.unam.mx; 3 Departamento de Ingeniería Bioquímica, Instituto Politécnico Nacional, Mexico City 07700, Mexico; jhernandezv1717@alumno.ipn.mx; 4 School of Engineering and Sciences, Tecnologico de Monterrey, Queretaro 76130, Mexico; thomas.stringer@tec.mx (T.S.); monserrat_ramirez@tec.mx (M.R.-M.); 5 Department of Analytical and Environmental Chemistry, Southwest Research Institute, 6220 Culebra Road, San Antonio, TX 78238, USA; alice.yau@swri.org

**Keywords:** bio-based elastomer, rubbers, polyurethane, polyester, polyether, elastomeric properties, olefin metathesis, circular economy, sustainability

## Abstract

In 2021, global plastics production was 390.7 Mt; in 2022, it was 400.3 Mt, showing an increase of 2.4%, and this rising tendency will increase yearly. Of this data, less than 2% correspond to bio-based plastics. Currently, polymers, including elastomers, are non-recyclable and come from non-renewable sources. Additionally, most elastomers are thermosets, making them complex to recycle and reuse. It takes hundreds to thousands of years to decompose or biodegrade, contributing to plastic waste accumulation, nano and microplastic formation, and environmental pollution. Due to this, the synthesis of elastomers from natural and renewable resources has attracted the attention of researchers and industries. In this review paper, new methods and strategies are proposed for the preparation of bio-based elastomers. The main goals are the advances and improvements in the synthesis, properties, and applications of bio-based elastomers from natural and industrial rubbers, polyurethanes, polyesters, and polyethers, and an approach to their circular economy and sustainability. Olefin metathesis is proposed as a novel and sustainable method for the synthesis of bio-based elastomers, which allows for the depolymerization or degradation of rubbers with the use of essential oils, terpenes, fatty acids, and fatty alcohols from natural resources such as chain transfer agents (CTA) or donors of the terminal groups in the main chain, which allow for control of the molecular weights and functional groups, obtaining new compounds, oligomers, and bio-based elastomers with an added value for the application of new polymers and materials. This tendency contributes to the development of bio-based elastomers that can reduce carbon emissions, avoid cross-contamination from fossil fuels, and obtain a greener material with biodegradable and/or compostable behavior.

## 1. Introduction

According to PlasticsEurope AISBL©, the global plastics production data in 2021 was 390.7 Mt, and in 2022, it was 400.3 Mt; where Africa produced 9%, Europe 14%, America 21%, and Asia 56%, with China being the country with the highest plastic production, with about 32%. These include conventional plastics and thermoplastics such as PET, PE, PP, PVC, PS, and PU, as well as some thermoset plastics [1,2]. The production and consumption of conventional plastics and polymers show an increase year after year. 

Elastomers, a category of polymers characterized by high elasticity and viscoelasticity, possess the ability to revert to their initial form after undergoing stretching or deformation and are known for their outstanding resistance to abrasion, tearing, and impact. Elastomers find applicability across various industries, including automotive, aerospace, construction, electronics, healthcare, and biomedicine [3,4,5]. There are two types of elastomers: thermoplastic elastomers (TPEs) (e.g., thermoplastic polyester elastomers, thermoplastic polyether elastomers, thermoplastic polyurethane elastomer, thermoplastic polyamide elastomers) and thermosets (e.g., vulcanized natural or industrial rubber, epoxidized natural rubber, cross-linked polyester elastomers, cross-linked polyurethane elastomers). Thermoset elastomers predominate in production and consumption. The elastomer industry encompasses three primary kinds: rubber, plastic, and gel. Rubber elastomers, crafted from natural or synthetic polymers, are renowned for their remarkable elasticity and durability. In contrast, plastic elastomers or TPE represent a fusion of rubber and plastic, delivering a harmonious combination of flexibility and rigidity [3,4]. Currently, polymers, including elastomers, are non-recyclable and come from non-renewable sources [5]. Due to this, the synthesis of elastomers from natural and renewable resources has attracted the attention of researchers and industries. 

By the definition provided by the International Union of Pure and Applied Chemistry (IUPAC), the term “bio-based” refers to “materials that are either entirely or partially composed of biological products originating from biomass. This encompasses materials derived from plant, animal, marine, or forestry sources” [6]. In environmental terms, bio-based polymers possess the capacity to mitigate greenhouse gas (GHG) emissions, particularly carbon dioxide (CO_2_). This reduction occurs because, during the synthesis process using natural resources as raw materials, plants absorb atmospheric CO_2_ for their growth. As a result, bio-based polymers contribute to a net reduction in atmospheric CO_2_ levels [7].

A bio-based polymer originates from natural and/or renewable sources, typically derived from biomass, and is recognized for its environmentally friendly characteristics. It exhibits a shorter environmental decomposition time, influenced by both abiotic and biotic factors [8]. This polymer or elastomer may consist partially or entirely of bio-based components, such as a mixture, composite, copolymer, graft, additive, or by employing natural monomers in the synthesis (biopolymer). A higher percentage of bio-based content indicates a more sustainable material, often associated with biodegradable and compostable behavior [9].

In this review paper, new methods and strategies are proposed for the preparation of bio-based elastomers. The main goals are the advances and improvements in the synthesis, properties, and applications of bio-based elastomers from natural and industrial rubbers, polyurethanes, polyesters, and polyethers, and an approach to their circular economy and sustainability.

## 2. Bio-Based Elastomers from Natural and Industrial Rubbers

Synthetic elastomers used in tires are derived mainly from petrochemicals, which are not sustainable. Recently, renewable resources have been used to produce several bio-based elastomers. For example, itaconic acid is a renewable resource from fermentations produced by microorganisms, especially *Aspergillus terreus*; it is used as a bio-based building block. Lei et al. have manufactured silica/poly(di-*n*-butyl itaconate-co-butadiene) (20–80% butadiene) nanocomposite-based green tires that are prepared by redox-initiated copolymerization of di-*n*-butyl itaconate and butadiene. These bio-based elastomers have excellent wet skid and low roll resistances, respectively [10]. 

It is essential to say that not all bio-based polymers are biodegradable (capability of being degraded by biological activity); for example, bio-based plastics such as the bio-polyethylene (Bio-PE) and Bio-poly(ethylene terephthalate) (Bio-PET) that use bioethanol from sugarcane (by the fermentation of glucose) [8,11,12], starch-based crops produced by wet-mill processing as raw material [13,14], the chemical modification of natural rubbers (NR) that have been modified to improve their properties and application, as well as the epoxidized NR under strain-induced crystallization (SIC) [15], are all non-biodegradable polymers.

In particular, natural rubber (NR) is a polymer composed of isoprene units linked to form double bonds with a *cis* configuration (99.9%, *cis*-1,4-polyisoprene) when extracted from the latex of the *Hevea brasiliensis* trees and *Parthenium argentatum woody shrub* [16,17,18]; *trans* configuration extracted from the latex of the *Palaquium gutta* trees (*trans*-1,4-polyisoprene) [17,19]; and *cis*/*trans* isomer mixtures extracted from the latex of the *Manilkara zapota* trees [17,18,20]. NR (99.9%, *cis*-1,4-polyisoprene) is widely employed in the fabrication of tires, construction industries, and biomedical applications. NR possesses unique properties due to its low modulus and high elasticity and can rapidly recover its original state upon release of external stress [18]. 

**Figure 1:**
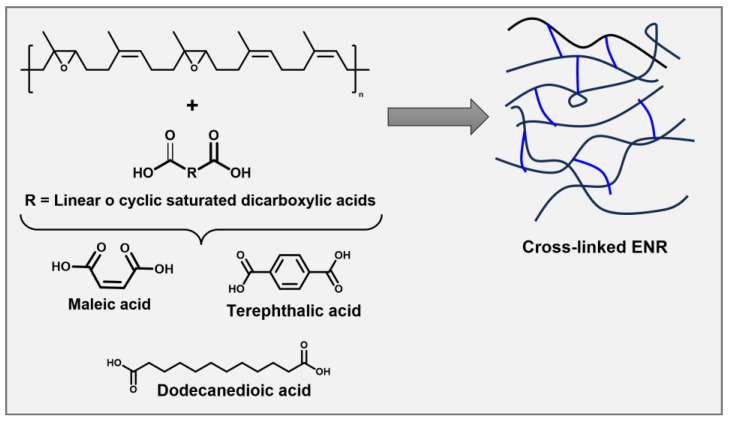
Crosslinked epoxidized natural rubber (ENR) with dicarboxylic acid.

Based on reprocessability, bio-based NR can introduce bio-based chemically crosslinked thermoplastics. The bio-based chemically crosslinked/cross-linkable elastomers (CCEs) from NR and synthetic elastomers derived from bio-based monomers have been reported. CCEs possess better resilience and higher operating temperatures due to covalent three-dimensional (3D) networks [4,21,22,23,24]. Several dicarboxylic acids are used as green crosslinkers to react with ENR (Figure 1).

Srirachya et al. investigated a crosslinking agent for ENR. They demonstrated that it was possible to crosslink ENR with maleic anhydride thermally; no additional catalysts were needed [21]. Riyajan et al. studied the influence on the physical properties, including the swelling behavior, tensile strength, and thermal stability of the vulcanization of ENR latex using a terephthalic acid crosslinker. It was observed that the ENR crosslinked with terephthalic acid depolymerized in natural soil compared to natural rubber vulcanized with sulfur [22]. Pire et al. crosslinked the epoxidized NR by dodecanedioic acid with 1,2-dimethylimidazole. The authors explain that 1,2-Dimethylimidazole accelerates crosslinking, and imidazolium dicarboxylate species are formed to reach the less substituted side of the epoxy site [23,24]. Generally, the ENR crosslinked with dicarboxylic acids improves mechanical properties compared to those vulcanized by sulfur because carbon–oxygen crosslinks possess higher bond energies than sulfur ones [25].

### Bio-Based Elastomers with Essential Oils

**Figure 2:**
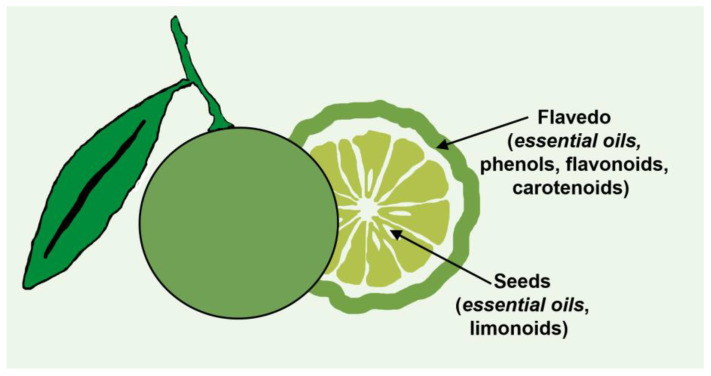
Essential oils in the citrus peels and cuticle of the citrus fruit.

Citric fruits (*Rutaceae family*) are the most commonly cultivated and consumed fruits worldwide. According to the Food and Agriculture Organization of the United Nations (FAO), global overall citrus fruit production in 2020 was around 158.49 million metric tons [26]. The major citrus-producing countries include China, Brazil, and the United States of America. Approximately 40–60% of the fruit is discarded as waste upon consumption. In general, the industrial production of citrus fruits generates 110–120 million tons of citrus waste yearly, such as peels (flavedo and albedo), seeds, and pomace [27,28,29]. It is possible to extract limonoids, essential oils, phenols, flavonoids, carotenoids, and cellulose from these wastes. Significantly, essential oils can be obtained from the peels and seeds of citrus fruit waste, and they are considered potent bio-resource materials for various uses in the food and non-food sectors. Also, these substances are considered plant-based natural products consisting of volatile and aromatic compounds present at low concentrations. Essential oils are compounds made up of isoprene units attached to 10-carbon and 15-carbon structures known as monoterpenoids and sesquiterpenoids, respectively. These compounds are typically found in the oil sacs of citrus peels and cuticles, as shown in Figure 2 [29]. 

Essential oils or citrus oils have the characteristics of being low in toxicity, inexpensive, and can be obtained by several extraction techniques such as steam distillation and solvent extraction [30,31]. They are mainly used in the pharmaceutical industry to obtain active ingredients, the cosmetics industry in perfume production, food for flavoring preparation, and the chemical industry in packaging materials production, among others. In the bio-based elastomers elaboration, essential oils have significantly modified natural rubber and some industrial elastomers.

Terpenes have emerged as viable candidates to serve as monomers in bio-based elastomer production. The most common type of bio-based elastomers from terpene is β-Myrcene, which is extracted by hops, bay, and thyme oils or can be obtained by pyrolysis from β-pinene [32]. β-Myrcene has two conjugated double bonds in its structure and can be polymerized similarly to its isoprene via anionic, free-radical, coordination, and cationic reactions. These polymerization reactions can have several possibilities for 1,2-; 3,4-; and 1,4-*cis* or t*rans* myrcene isomerization to obtain elastomers with similar mechanical and thermal properties to NR (*Hevea brasiliensis, Palaquium gutta*, and *Manilkara zapota* trees). In 1960, Marvel studied the polymerization of myrcene by Ziegler-type catalysts (triisobutylaluminum, TiCl_4_, and vanadium trichloride) with a predominantly 1,4 arrangement of the diene unit in the polymer chain, with conversions of 80% of the polymyrcene [33].

It has been reported the stereoselective polymerization of β-myrcene and β-ocimene and their copolymerization with styrene using homogeneous titanium catalysts activated by MAO (methylaluminoxane) showing different stereoselectivity, for example, 1,4-*trans*-poly myrcene (92%) and its copolymerization with styrene using dichloro{1,4-dithiabutanediyl-2,2′-bis(4,6-di-tert-butyl-phenyl)}titanium complex, and 1,4-*cis*-poly myrcene (92%) in the presence of Ti(η5-C_5_H_5_)-(η2-2,2′-methylene bis(6-tert-butyl-4-methylphenoxo))Cl complexes were obtained. Also, these catalysts were used in the β-ocimene polymerization. Other terpenes are ocimene (which can be produced from the thermal cracking of β-pinene) and farnesene (which can be found in nature like α- and β-isomers); however, farnesene can be obtained through the fermentation of sugar feedstocks [34]. When using the last catalysts with ocimene and a high temperature (70 °C), the 1,4-*trans* polymer (70%) is formed, while at a lower temperature, an isotactic poly-1,2-ocimene (>99%) was obtained [35].

On the other hand, Lamparelli et al. have reported that the soluble heterocomplexes consisting of sodium hydride in combination with trialkylaluminium derivatives have been used as anionic initiating in the polymerization of myrcene and its copolymerization with styrene and isoprene [36,37].

It is well known that NR and industrial elastomers such as polybutadiene (PB) and poly(styrene-butadiene-styrene) (SBS) are materials used in the production of tires, adhesives, and rubber bands, among others. The properties of elastomers are amorphous polymers found above their glass transition (Tg), which explains their deformability. Authors have demonstrated that modified elastomers such as NR are proposed to expand the application [38]. It has been reported that *d*-limonene, β-pinene terpenes, some menthol, β-citronellol, and citral terpenoids, mainly extracted from turpentine, lemon, mandarin, orange, mint, citronella oils, citral, and other terpenes, contain in their structure carbon–carbon double bonds, and they are used in the natural rubber modification via metathesis reactions [39,40,41,42].

**Figure 3:**
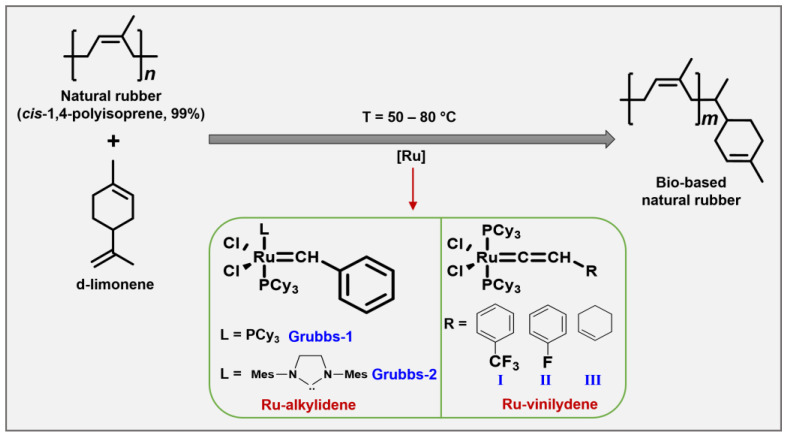
Synthesis of bio-based NR with d-limonene via *cross*-metathesis.

Some reports have shown that *d*-limonene, considered the most abundant component in mandarin (74%), lemon (87%), and orange (97%) essential oils, is used as a chain transfer agent (CTA) in the *cross*-metathesis reaction of NR in the presence of ruthenium carbene-alkylidene and vinylidene complex catalysts (Figure 3). These two classes of catalysts tolerate a wide range of functional groups (e.g., amino, carboxyl, acetoxy, ester, hydroxy, fluorine, and chlorine), and they are active with more sterically hindered substrates such as natural rubber [43,44,45].

NR modification with limonene and mandarin oils via *cross*-metathesis allows the synthesis of mono-terminated or terpene-terminated oligomers (Table 1) with low molecular weight around *M_n_* × 10^2^ g × mol^−1^ and yields of 80% when the catalyst Grubbs-2 is used (*entries 1 and 3*). In contrast, with Grubbs-1 and vinylidene-II and -III catalysts [40], the molecular weights of terpene-terminated oligomers are around *M_n_* × 10^4^ g × mol^−1^ with yields ranging from 70–96% (*entries 2, 7, and 8*).

It essential to note that Ru-vinylidene catalysts (I) showed higher catalytic efficiency in the *cross*-metathesis reaction of NR with d-limonene and mandarin oil to give terpene-terminated oligomers with *M_n_* × 10^3^ g × mol^−1^ (*entry 6*) [41] Also, authors reported that *cross*-metathesis reaction can control the molecular weight by the NR/CTA molar ratio (*entries 3–5*). A similar approach, bio-based NR, was obtained using lemon, orange, and β-pinene terpene as CTAs [39,40,41,46]. This easily accessible class of Ru complexes showed high thermal stability combined with good activity in the NR *cross*-metathesis.

Theoretical studies have shown that trisubstituted internal olefin (NR, d-limonene) is more difficult to break the C=C bond than one disubstituted unsaturation of the *cis*-polybutadiene and other polyalkenamers due to the steric hindrance of the methyl group of the NR, which makes difficult the coordination between the M=C complex and C=C double bond of isoprene [47]. For example, this has been confirmed by experimental studies in the *cross*-metathesis of SBS synthetic elastomer using d-limonene and orange oil as CTA. The SBS-terpene elastomer modification in the presence of ruthenium–vinylidene catalysts (I) and Grubbs-1 to several molar ratios [rubber]/[CTA] was accomplished.

## 3. Bio-Based Polyurethane Elastomers

The global production of polyurethane (PU) remains between 5.3 and 5.5% compared to conventional plastics such as PET, PE, PP, PVC, and PS [1,2,8]. PU has gained much attention due to its excellent thermal, mechanical, and chemical properties, diversity in material types, and their applications. There are different types of polyurethanes, including thermoplastic polyurethanes (TPUs), thermostable polyurethanes, and elastomeric polyurethanes (EPUs). The most common thermostable polyurethanes are foams, widely used as thermal insulators; among the most common EPUs are those used as adhesives, high-performance sealants, shoe soles, paints, textile fibers, gaskets, and in the construction, furniture, medical, and automotive industries. The characteristic properties of elastomeric PU include high resistance to wear and abrasion, good cushioning capacity, good flexibility at low temperatures, high resistance to fats, oils, oxygen, and ozone, excellent elastic recovery, transparency, and adhesion [48,49,50,51].

PU is synthesized by a polycondensation reaction between a diisocyanate and polyol or macrodiol with an average molecular weight in the range from 600 to 4000 g/mol, using a short-chain diol or chain extender with molecular weights in the range of 62 to 400 g/mol to increase the chain’s molecular weight and define the material’s rigidity. The polyol forms the soft and flexible segments and provides an elastomeric matrix responsible for most of the elastic properties of EPU. The diisocyanate, polyol, and diol are used at different molar ratios, and the reaction mixture will define the soft and hard segments, the type of PU, its properties, and its applications [49,52,53,54].

These three components or raw materials for the production of PU are derived from oil resources. Currently, PU is a non-recyclable polymer that comes from non-renewable sources. It can also be a thermoplastic PU, which makes it a complex material to recycle and reuse; moreover, there is no adequate waste management. It takes hundreds to thousands of years to decompose or biodegrade, contributing to plastic waste accumulation, nano and microplastic formation, and environmental pollution [55,56]. Due to this, the synthesis of PU from natural and renewable resources has attracted the attention of researchers and industries to form different types of bio-based PU [51,57,58,59]. Precisely for this study, bio-based EPU shares elastic properties like rubbers and thermoplastic polyurethane; these properties are provided mainly by the polyol from natural resources, making it ideal for different applications. 

**Figure 4:**
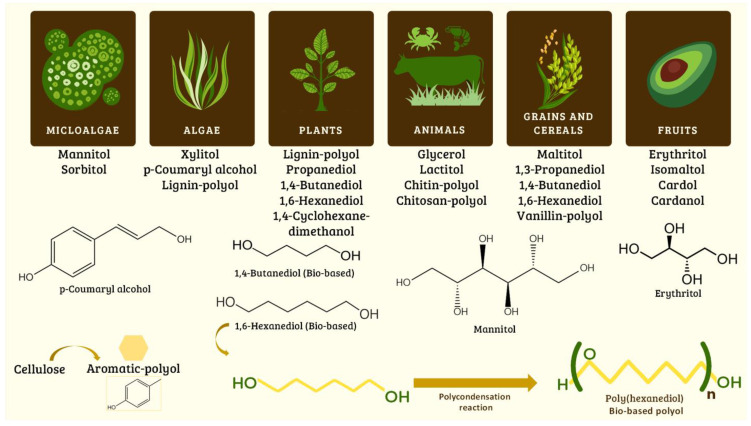
Synthesis of bio-based diols and polyols from natural and renewable resources used to synthesize polyurethanes.

Different diols and polyols have been used to synthesize bio-based EPUs derived from algae, plants, animals, crustaceans, grains, cereals, and fruits. Some diols and polyols are mannitol, sorbitol, xylitol, glycerol, cardol, 1,3-propanediol, 1,4-butanediol, and 1,6-hexanediol bio-based (Figure 4). Many polyols have been obtained and modified from lignin, chitin, chitosan, and cellulose (aromatic polyol) [60,61,62,63,64,65,66]. Some diols can be converted into polyols by a polycondensation reaction; for example, polyhexanediol (bio-based polyol) has been obtained from 1,6-hexanediol [62]. 

Various polyols have been synthesized from industrial and natural rubbers, which have elastomeric properties because they come from rubbers, such as excellent elastic recovery, low transition temperature (Tg), and flexibility at low temperatures, which will be transmitted to the polyurethane. Polyols from polybutadiene (PB), polybutadiene rubber (BR), or SBS copolymer have been synthesized. BR has repeating butadiene units, which can be *trans* or *cis*-1,4-polybutadiene and are modified with another compound or reagent with hydroxyl (-OH)-terminated groups, which are called “hydroxy-terminated polybutadiene” (HTPB), hydroxy telechelic polybutadiene or polyols; these have been obtained by different methods such as oxidation or oxidolysis [67,68], anionic polymerization [69], via copolymerization [70], via nickel catalyst [71], and by olefin metathesis [72,73]. Other polyols have been obtained from natural rubber (NR), NR comes from the rubber tree, or from its synthetic form, polyisoprene rubber (IR), which has repeating isoprene units and can be *trans* or *cis*-1,4-polyisoprene [18]. In the same way, NR is modified with another compound with -OH groups, which are called “hydroxy-terminated polyisoprene” (HTPI or HTNR) or polyols; these have been obtained by different methods such as epoxidation [74,75], anionic polymerization [76], and by olefin metathesis [42].

**Figure 5:**
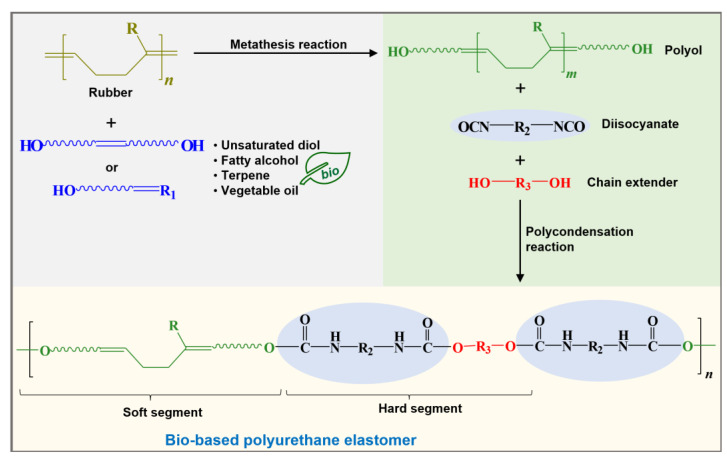
Synthesis of bio-based polyurethane elastomer from polyols obtained by industrial or natural rubbers degradation (providing the elastomeric part) and essential oils or fatty alcohols derived from natural resources. R is for rubber repetitive unit (R = H for PB, BR; R = CH_3_ for PI, IR, NR). R_1_ is for vegetable oil, unsatured diol, fatty alcohol, or terpene (e.g., 9-decen-1-ol, 10-undecen-1-ol, castor oil (*Ricinus communis*), citronellol, geraniol, myrcenol, eugenol). R_2_ is for aromatic or aliphatic structures for diisocyanates (e.g., MDI, TDI, IPDI, HDI). R_3_ is for C_2_–C_6_ chain extender length (e.g., ethanediol, propanediol, butanediol, hexanediol).

Of the mentioned methods, obtaining polyols by olefin metathesis reaction has shown that both natural and industrial rubbers can be modified or degraded under mild conditions, at low temperatures and atmospheric pressure, in solvent-free conditions, or allowing the use of green solvents, and using fatty alcohol, terpenes, diols, or essential oils with OH groups from natural resources such as chain transfer agent CTA for the synthesis and production of bio-based elastomeric polyurethanes (Figure 5). This is a new and promising synthesis route to produce bio-based polyols for applications in engineering polymers and elastomeric materials.

## 4. Bio-Based and Biodegradable Polyester and Polyether Elastomers

Since the last decade, developing new elastomers has brought infinite possibilities using natural materials for their synthesis. This new trend has arisen due to the high demand for elastomers from the market derived from non-renewable sources, which produces a constant negative effect [4]. These effects cause a series of environmental concerns. Throughout the entire technological production process and obtaining raw materials derived from oil, endless red dots cause extensive damage to ecosystems [77,78]. This environmental issue and the long degradation time have many unprecedented negative ecological consequences.

**Figure 6:**
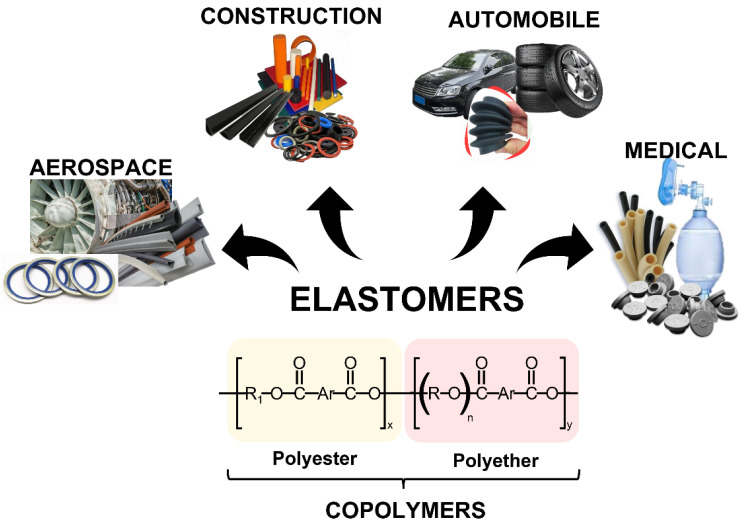
Some applications of polyester and polyether elastomers are used as copolymers. R and R1: alkylene; Ar: aromatic ring.

Based on those statements, the advantages of looking for new sources and routes to obtain elastomers continue to increase. Indeed, polyester and polyether arise as new sources of elastomers with many properties, starting from elasticity, which is the ability to recover the shape after a deformation [4,5]. However, to achieve this elasticity, the elastomers must have an elastomer and an elastic region over a considerable strain (Figure 6). When the elastomer has a rigid and flexible region, it is called a copolymer, and some particularities are involved in this type of material. Despite the viscoelasticity and weak inter-molecular forces, elastomers generally have low Young’s modulus but high failure strain. These peculiarities became elastomers as a tremendous and essential material for numerous applications such as aerospace, construction, automobile, and, more recently, biomedical industries [4,79,80]. 

Based on the type of crosslinking, elastomers can be divided into main groups: the chemically crosslinked and the physically crosslinked. The last one has a more thermoplastic function, and it is named physically crosslinked thermoplastic elastomers (TPEs). It is well known that chemically crosslinked elastomers possess excellent resilience and higher operating temperatures since the covalent functionality allows the creation of 3D networks in the material. This type of elastomer is commonly known as “rubbers” and their applications will be limited depending on the additives used to obtain a particular elastomer for further engineering applications [81,82]. 

Since natural and synthetic rubbers have a global consumption of more than 14 Mt in 2022 [73], the TPEs became more important since they possess a microphase-separated structure, avowing the use of reinforced fillers. This particularity is essential in terms of the recyclability of the material. Still, commercial synthetic elastomers are produced or obtained from fossil resources, translating into more environmental problems than natural rubbers or other elastomers. Indeed, researchers’ primary objective is to develop bio-based elastomers to reduce carbon emissions, avoid cross-contamination from fossil fuels, and obtain a greener material with biodegradable and/or compostable behavior. 

### 4.1. Generalities from Polyester and Polyether Elastomers

Based on the chain chemistry of the elastomer, these materials can be divided into polyester elastomers and polyether elastomers, depending on the segment of the general formula. However, segmented copoly (ether-ester) elastomers are also included in the group of ester elastomers. Several authors reported a synthesis course in which phase separation occurs when the substituted (normally polyols) molecular weight is high enough. Then, a soft phase with a noticeable low glass transition temperature and a hard phase with a high melting point are designed [4,79]. Until now, segment modifications have started to include bio-based materials, but usually, the elements joining polyether and polyester segments could be ester, amide [83], urethane [84], and ether groups [85]. 

In general, these elastomers can be easily synthesized through an esterification and polycondensation process. Meanwhile, changing the ratios and varying the compounds used as hard and soft segments can improve their physical properties. These characteristics bring a thermodynamic immiscibility material in which the rigid phase is separated from the flexible segments (phase separation), and two phases are found in the material, a softer one with different glass transition and melting temperature than the rigid phase [77]. In terms of composition, the hard segments are generally aromatic polyesters, while the soft segments are aliphatic polyethers or polyesters with long chains. Some examples are found in the literature for hard segments, which include poly(trimethylene terephthalate) (PTT), poly(butylene terephthalate) (PBT), and Polyether ether ketone (PEEK) [86]. In the case of soft segments, polyol molecules such as poly(ethylene glycol) (PEG), poly(tetramethylene glycol) (PTMG), and poly(sebacate ethylene glycol) are a few of those used in the formulation [87]. The most common strategy for synthesized and fabricated polyester and polyether elastomers is the introduction of long aliphatic flexible soft segments into the most rigid phase material. This process gives the polyester–polyether elastomers a range of unique properties, such as similar synthetic features as polyesters, but biodegradation is still missing. 

For this reason, efforts to find alternatives to more environmentally friendly materials have been studied. Indeed, other polyols, such as natural and vegetal oils, glycerol and its derivatives, terpenes, and resins, synthesize copolymers to obtain excellent polyesters and polyether elastomers. The idea of producing these plastics is to proceed with linear degradation, which means that only at the surface of the material exposed to water are mass loss and dimensional reduction proportional to the surface area exposed. 

This process can be enhanced if a polyester or polyether is included in the elastomer structure, increasing the mechanical properties but enhancing its performance in terms of thermal stability, chemical resistance, and structural integrity. To understand this enhanced mechanism deeply, Table 2 shows some examples of bio-based and biodegradable polyester and polyether elastomers found in the literature and their respective characteristics.

### 4.2. Bio-Based and Biodegradable Polyester/Polyether

To understand the biggest challenges for formulating bio-based and/or biodegradable polyester/polyether elastomers, some researchers plan experimental strategies in Table 2.

Remarkably, the strategies can be divided into groups depending on the bio-based or/and biodegradable source in which the monomer for polymerization is presented. Depending on the applications, different additives or rigid polymers are included to enhance their properties. Indeed, Groeneveld [88] used the exceptional characteristics of the Castor plant to extract ethoxylated oils, promising sources of mono-, di-, tri-, tetra-, and penta-esters using glycerol derivates. The authors founded a technique based on two-dimensional liquid chromatography hyphenated with high-resolution mass spectrometry to obtain polyether(ethers) with polyols, which can be used as initiators for the synthesis of polyurethanes. Since the techniques are valuable for the comprehensive separation of overly complex, the uses for this type of components are variable, from coatings, adhesives, sealants, and synthetic lubricants to functional fluids in which isomers are pretty necessary. 

Another example using plant-derived materials is the one presented by Martínez et al. [89], in which the use of polyterpene natural rubber from *Hevea brasiliensis* was implemented in a *cross*-metathesis reaction with α,β-unsaturated carbonyl compounds. 

Authors claim that preparing unsaturated polyester elastomers has excellent potential as a petrochemical alternative, including in perfume, flavor, and pharmaceutical industries. These results were conclusive when novel aliphatic unsaturated esters with molecular weight values around Mw = 1912 to 3367 g/mol and yields ranging from 68 to 79% were obtained. This result is encouraging since terpenes from plant-derived can be used satisfactorily in this type of chemical reaction. 

Similar to the previous results, residues from plant-based biomass rich in hemicellulose can be used to form α,ω-unsaturated polyesters and polyether. To get this, Piccini et al. [90] use a bicyclic diol 1,2-O-isopropylidene-α-D-xylofuranose D-xylose derived from the biomass and a mixture with ω-unsaturated fatty acids and alcohols, originating a promising bioplastic for sustainable packaging applications. The authors employed an acyclic diene metathesis polymerization and found a decrease in the glass transition temperatures when increasing the chain length of the additives. Moreover, after being cast into films, the elastomers displayed remarkable polyethylene-like properties with high Young modulus and elongation at break, solid yet flexible behavior, and excellent gas barrier properties, proving their uses as food packaging materials. 

Indeed, the uses of plant-derived materials are getting increasingly valuable, and Linh et al. [91] presented the synthesis, preparation, and properties of some polyethers from chalcones. Chalcones (or 1,3-diphenyl prop-2-en-1-one) are promising molecules with α,β-unsaturated carbonyl groups [96]. Linh et al. [91] mainly use the 4-hydroxy benzaldehyde and vanillin with p-hydroxy acetophenone to synthesize diphenol chalcone-derived monomers. The Claisen–Schmidt condensation reactions with 1,6-dibromo hexane were performed for the elastomer formulation, and a co-polyether’s materials were achieved. Even when several applications in pharmaceutical compositions, microscopy, photosensitive materials, nonlinear optical materials, and crystal display liquids are found, the authors believe that further studies in the field of photochemistry and photosensitivity need to be performed. This hypothesis was based on the TGA analyses that proved that polymer samples had good thermal stability. 

Another biomass for the obtention of copolyether polyols is presented by Basterretxea et al. [77] when lignocellulosic and limonene terpenes are applied into *self-*condensation and polycondensation. The authors claim the preparation of ideal candidates in a great variety of polyurethane elastomers is due to the comprehensive properties and tunable crystallinity found in the materials. Since lignocellulosic materials and hemicelluloses are presented in many plant-derived materials, their uses are common in the obtention of polyester and polyether elastomers. Sousa et al. [87] use some natural sources such as ethylene glycol and 2,5-furan-dicarboxylic acid to produce the poly(ethylene 2,5-furan-dicarboxylate) and poly(ethylene glycol) elastomers by polytransesterification reaction. These furandicarboxylated copolyester/ethers are essential insights for the packaging industry since lower melting temperature and higher thermal stability were found in other investigations by Qu et al. [94] for the preparation of bio-Furan-based polyesters in food and beverage packaging, clothing, and in the car industry. One type of biomass source with lignocellulosic components in corn, and Luo et al. [93] presented the synthesis and evaluation of polyether diols-based polyurethane dispersions by a solvent-free process using poly propanediol and poly(tetramethylene ether) glycol as copolymer. The solvent-free method was enough to obtain a polyether diol-based polyurethane that can be included in textile coatings since it presents high adhesion ability and excellent softness.

Finally, it is essential to consider that other renewable resources such as polylactic acid (PLA) [92] and citric acid [97] are still undergoing investigation to be considered excellent components to produce polyester and polyether elastomers. Indeed, Nagarajan et al. [92] evaluated the production of a thermoplastic copolyester elastomer using PLA and Hytrel, a commercial thermoplastic polyester elastomer, to produce a more environmentally friendly material based on the substitution of the bio-based Hytrel into several compositions with the PLA being the optimal the formulation with 70/30. Hytrel is a polyether glycol and polybutylene terephthalate elastomer, but when glycidyl methacrylate, maleic anhydride, and a melting processed reaction using ethylene butyl acrylate and ethylene methyl acrylate copolymers, some properties such as a co-continuous morphology and changes in tensile strength and modulus of the binary blends decreased depending on the content of Hytrel. However, impact toughness and a more significant stress whitening zone were observed in the impact-fractured composite surface with ethylene methylene acrylate-glycidyl methacrylate, showing better results. This result is interesting when reducing bio-based materials from non-renewable sources is included in producing sustainable copolymers for commodity and engineering applications.

Similarly, Acosta et al. [95] studied substituting or reducing a commercial greenpoxy 28 material obtained from plant and vegetable origin into a reinforced composite. The authors explain that it is possible to treat the composite to reduce its amount in the preparation of elastomers. For this, the woven jute fabric is used as reinforcement material and allyl-functionalized ditertiary amine as a curing agent and a multifunctional thiol (3-mercaptopropyl (trimethoxysilane)) as a radical photoinitiator. The authors prepared the blends using epoxy/thiol-ene photopolymerization to obtain a polyether-polythioether crosslinked co-network. The results indicate an increase of up to 50% in flexural modulus and strength concerning the non-functionalized counterparts. Also, the composites displayed higher deformation at break and toughness due to the presence of polythioether in the co-network. The prepared biocomposites could be excellent material applications in aerospace, nautical, automotive, packaging, and building industries.

### 4.3. Advances in the Use of Bio-Based and Biodegradable Polyester and Polyether Elastomers

**Figure 7:**
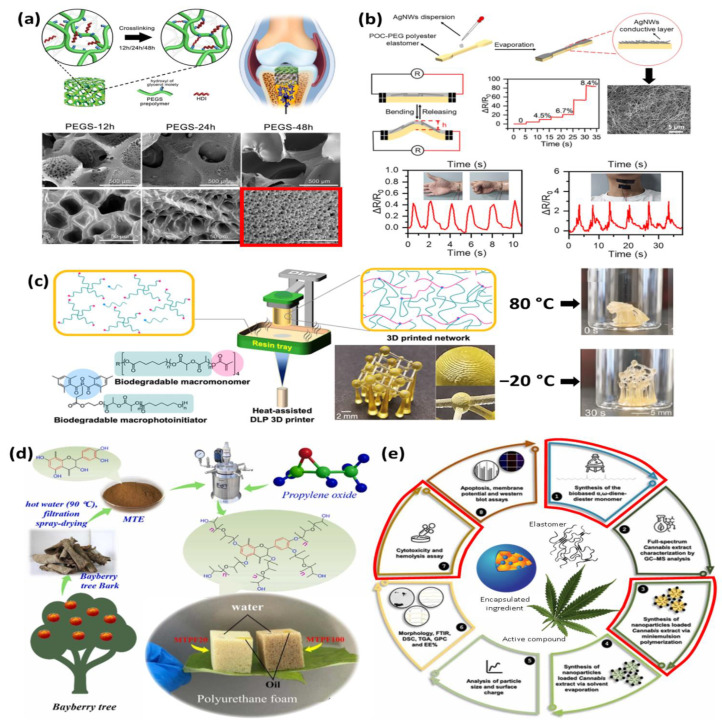
Future of bio-based elastomers applications for (**a**) biomedical (reprinted/adapted with permission from Ref. [84], 2023, Elsevier), (**b**) sensors (reprinted/adapted with permission from Ref. [97], 2023, OAE), (**c**) bone reconstruction (reprinted/adapted with the permission from Ref. [98], 2023, ACS publications), (**d**) advanced materials (reprinted with permission from Ref. [99], 2023, Elsevier), and (**e**) drug delivery (reprinted/adapted from Ref. [100], 2023, Elsevier).

Although the advances in the search for bio-based or biodegradable elastomers continue to increase, the expectations for using materials from polyester and polyether elastomers continue to be infinite. Various authors describe that polyester or polyether elastomers are excellent candidates in terms of durability, degradability, costs, and synthesis; however, many studies only focus on the chemical formulation and its characterization, and few studies explain and demonstrate with a straightforward application, concise and precise, the improvements and virtues of the new composites created. Thus, a series of articles were selected because their applicability in biomedical, sensors, reconstruction, advanced materials, and drug delivery (Figure 7) are clearly explained, and they constitute an excellent example to continue with the development of said materials.

In the biomedical area, Lin et al. [84] prepared polyethylene glycolated (PEG) poly(glycerol sebacate) scaffolds employing solvent-free urethane crosslinking and spontaneous pore-forming procedure at room temperature (Figure 7a). The authors include a hexamethylene diisocyanate (HDI) crosslinker to evaluate the controllable crosslinking degrees and hierarchical macro-/micro-porosities. Also, a bilayer with an osteoinductive mesoporous bioactive glass (MBG) could enhance the reconstruction in vivo assessments. Authors found that the experiment, conducted for at least 12 h, reveals a low crosslinking degree which stimulates chondrogenic differentiation, maintains chondrocyte phenotype, and enhances cartilage matrix secretion compared to elastic polymer with a high crosslinking degree, emphasizing the importance of matrix viscoelasticity in cartilage regeneration. This proposal for osteochondral regeneration is an excellent candidate and was expected for clinical translation to exhibit extraordinary regenerative efficiency after 12 weeks in the subchondral bone. Moreover, Wang et al. [97] presented degradable citric acid-based conductive polyester elastomers in terms of platforms for sensing. The platform created after the polycondensation process with citric acid, 1,8-octane diol, and poly(ethylene glycol) as monomers were coated with a silver nanowire layer and soft electronic material for flexible electronic devices was created. The mechanical stimuli evaluation of the poly(1,8-octane diol citrate acid) (POC) copolymer with PEG exhibits recoverable deformation after bending, converting strain to detectable resistance. For the sensing evaluation in humans, various human emotions were successfully evaluated with a wireless sensor. The results demonstrated that degradable citric acid-based polyester elastomers are promising for next-generation sustainable and flexible electronic devices.

In the same field of biomedicine, Sandmeier et al. [98] presented a biodegradable elastomer obtained by ring-opening polymerization of phenyl bis (2,4,6-trimethyl-benzoyl)phosphine oxide (BAPO) with 2-hydroxyethyl acrylate (HEA) or 2,3-hydroxypropyl acrylate (DIOL). The well-defined photoinitiator-polymer conjugates BAPO-HEA and BAPO-DIOL were digitally light-processed to obtain a 3D printing material without organic solvents. The result indicates that photopolymerization provides new opportunities for fabricating tissue scaffolds and medical devices with high-quality 3D printing in biodegradable shape-memory devices. Also, the final material exhibits this shape-memory behavior after the temperature is applied. The initial object was deformed at ~80 °C and fixed in that shape by cooling to −20 °C. This work offers new perspectives for the solvent-free additive manufacturing of bioresorbable medical implants and other functional devices. In the same order, Zhang et al. [99] presented an advanced material based on the design and manufacturing of cost-effective tannin-based polyether polyol (MTEBP) that was synthesized using tannin extract (MTE) as an efficient and reusable absorbent for oil and solvents. After copolymerization, the authors evaluated the adsorption capacities of the materials to soybean oil, xylene, dichloromethane, n-hexane, and cyclohexane, increasing their values by 53.5%, 57.9%, 115.1%, 83.5%, and 97.2%, respectively. Also, mechanical and thermal properties were enhanced after formulation. This work affords a feasible and sustainable strategy to fabricate bio-based, eco-friendly, tannin-based materials with high adsorption capacities for oil spill clean-ups.

Finally, a very new technique to implement biodegradable elastomers into a drug delivery system is presented by Freire et al. [100] (Figure 7e). This study aimed to develop bio-based poly (thioether-ester) nanoparticles containing full-spectrum cannabis extracts and assay their potential efficacy in vitro BF16F10 melanoma cells. Tetrahydrocannabinol, cannabidiol, and other natural compounds produced by *Cannabis sativa* exhibit a wide array of therapeutic effects on the human body but limited bioavailability due to their low solubility in water and moderate stability. The result indicates that poly (thioether-ester) nanoparticles were effective, and nanocarriers and potential anticancer activity could be demonstrated.

## 5. Elastomers: The Circular Economy and Sustainability

The current development of our society is only possible by considering the role of polymers or plastics [101]. The generation and disposal of synthetic plastics are among the major current concerns in the sustainability discourse of large industries. The current issues with elastomers in terms of sustainability are multifaceted and include resource dependency, energy-intensive production, end-of-life management, microplastic pollution, toxic additives, and a lack of circular economy practices [102,103].

In recent decades, efforts have been made to develop various types of biodegradable plastics, aiming to constitute a class of “green polymers” following the principles of green chemistry. However, the degradation of plastics in landfills does not lead to the recovery of material value, and their degradation in oceans could create new or unwanted environmental impacts [104]. Strategies have been implemented to promote reuse and recycling techniques, extending the lifespan of materials and promoting their integration into a circular economic model [105,106]. As such, a wide breadth of literature has touched upon circular principles in the context of elastomers. Different R-strategies have been proposed or analyzed, and themes related to sustainability have also been integrated into part of the studies. This section explains the concepts of a circular economy and sustainability and describes which circular strategies have been mentioned in the previous elastomer literature.

### 5.1. The Circular Economy

Circular economic processes are not new and have been used for thousands of years. However, the foundations of the concept in the literature are reasonably recent. According to Mohajan, the term “circular economy” gained prominence in the late 20th century as a response to environmental and resource challenges [107]. Stahel and Reday first describe a loop economy framework that describes waste reduction and resource efficiency strategies and emphasizes the need to design products for longevity and efficient resource use [108]. Pearce and Turner explore the relationships between natural resources in the economy and the linearity of traditional economic systems [109]. One of the most commonly used definitions, a product of the Ellen MacArthur Foundation [110], defines a circular economy as “an industrial economy that is restorative or regenerative by intention and design”. Since the 2010s, many definitions have been proposed [111], with most focusing on reducing, reusing, and recycling resources, known as the first of the “R-strategies”. Geissdoerfer et al. define the circular economy as “a regenerative system in which resource input and waste, emission, and energy leakage are minimized by slowing, closing, and narrowing material and energy loops. These terms can be achieved through long-lasting design, maintenance, repair, reuse, remanufacturing, refurbishing, and recycling”. As such, the circular economy promotes resource efficiency and seeks to decouple economic growth from resource depletion and waste generation. This approach has gained significance in the 21st century as societies and businesses increasingly recognize the need for more sustainable and responsible economic models in the face of environmental challenges and resource constraints [112].

### 5.2. Adding Sustainability to the Mix: Eco-Concept

While sustainability is a term commonly used in the scientific literature, government documents, and the media, its definition is often ambiguous [113,114]. This lesser inclusion is partial because sustainability is a concept that has evolved dramatically since the beginning of the second half of the 20th century. The term was mainly used in the 1960s and 1970s to refer to environmental issues [115]. As one of the first contributions to early sustainability discussions about the trade-offs between economic development, social equity, and environmental conservation, “Limits to Growth”, published in 1972 [116], explores the implications of exponential economic and population growth and resource depletion. In 1987, the Brundtland Report, a result of the World Commission on Environment and Development, led by Gro Harlem Brundtland, famously defines sustainability as “development that meets the needs of the present without compromising the ability of future generations to meet their own needs” [117]. More recently, Geissdoerfer et al., after reviewing several definitions for the term, define sustainability as “the balanced and systemic integration of intra and intergenerational economic, social, and environmental performance”. Definitions that highlight the three pillars of sustainability, also known as the triple bottom line, are some of which are the most used today [112]. Even though circular economic applications can be solutions to achieving sustainability, both concepts are only sometimes aligned in the literature. Few circular economic definitions are linked to sustainable development, and economic prosperity is usually presented as the concept’s primary goal [111]. Circular economic processes are applied for economic and sometimes environmental considerations, but rarely social ones [118]. This means that sustainability trade-offs are not considered more often than otherwise. The existing literature [111,112,118] underlines the importance of accounting for sustainability when evaluating circular economy applications.

### 5.3. Elastomers and Circularity

Many studies focus on designing plastics that are more circular by nature. In their study, Hong and Chen [104] analyze the significant advancements in recyclable “green polymers” or chemically recyclable polymers, focusing on the technical and environmental benefits obtained in developing reuse and depolymerization processes for the chemical recycling of polymers at the end of their lifespan. Zanchin and Leone [119] review recent developments in thermoplastic polyolefin elastomers (P-TPE), highlighting their mechanical flexibility, lightweight, easy processability, and recyclability compared to thermosetting rubbers. P-TPEs meet the requirements for a sustainable circular economy for the next generation of plastics, showing promise as smart materials with shape memory and self-healing properties, particularly in innovative food packaging and coatings.

Utrera-Barrios et al., in four separate papers, introduce elastomer redesign with the analysis of self-healing properties. The first paper [101] reveals the challenges that rubbers face in reprocessing, suggesting self-healing to automatically repair or restore damages, thereby increasing their lifespan and aligning them with the circular economy model. Self-repairing rubbers exhibit high healing efficiency and exceptional mechanical behavior, essential properties sought in sustainable materials. A second paper [120] develops new biodegradable elastomer compounds capable of self-repair using a blend of epoxidized natural rubber and polycaprolactone reinforced with alginates (salt group). This combination gives the material thermal self-healing capabilities, catastrophic damage restoration, and the ability to recover its mechanical properties, contributing to the circularity of elastomeric materials. A third paper [105] delves into crucial self-healing concepts in the automotive industry’s most widely used elastomeric matrices. They analyze the state of the art considering the nature of the elastomeric matrix (natural or synthetic), different curing mechanisms (extrinsic, intrinsic, and combinations), the chemistry underlying them, and mechanical performance. This review highlights the transformative impact of these concepts in revolutionizing the industry and paving the way towards a more sustainable future. Finally, a fourth [121] addresses promising biodegradable plant-based fillers, their biosynthesis, chemical properties, applications, reinforcing effects, and improvements in the performance of composite materials based on natural and synthetic rubber matrices in elastomeric compounds. Sustainable fillers such as quercetin, chitosan, lignin, and cellulose are considered.

Other authors focus more on recyclability. Kumawat et al. seek to understand the relationship between the retention of physical properties and the load of granulated rubber and particle size. They characterize four different sizes of rubber particles physicochemically, concluding that finer particle sizes result in better dispersion of granulated rubber particles, providing superior mechanical, extrusion, and wear properties. This suggests that reducing particle size can increase the volume of recycled product utilization [106]. Georgescu et al. use recycled rubber in a range of 10–50% by weight as a filler material, eco-friendly reinforcement material, active loads, plasticizers, vulcanizing agents, and antioxidants to obtain chloroprene rubber. Recycled rubber reduces the compound’s ecological footprint by reducing its mass and revealing properties of elasticity, hardness, and abrasion resistance, making it a viable option regarding raw material life cycle, sustainability, eco-efficiency, and economic efficiency [122]. Kouhi et al. propose a simple method to reuse recycled rubber (RR) in closed-cell elastomeric foams based on ethylene propylene diene rubber (EPDM). Results in rheometry, morphology, and mechanical behaviors indicate that using RR could be a viable alternative for manufacturing high-performance EPDM foams with improved hardness and resilience, serving as an ecological substitute for virgin products [123].

Samir et al. explore the production of biodegradable polymers through chemical treatment, microorganisms, and enzymes, focusing on their environmental impact and ease of disposal. They investigate using natural fibers such as kenaf fiber, hemp fiber, and abaca fiber as reinforcement materials in biocomposites, highlighting their properties and potential applications to enhance the bond between the polymeric matrix and natural fibers in composites [103]. They emphasize the potential of biodegradable polymers to achieve Sustainable Development Goals (SDGs), particularly in the medical industry and in reducing the carbon footprint of plastic production. The proportion of ground tire rubber (GTR) in thermoplastic elastomers has also been analyzed as a circular economy strategy and sustainable development in the recycling of waste tire rubber. The results obtained differed from what was expected, as mechanical properties and processability decreased with increasing GTR content. The results improved by adding PE-g-MAH to the composition, particularly in tensile strength and elongation at break [124]. Gregory and Williams investigate the recyclability of thermoplastic elastomers (TPE) that do not degrade during recycling. They use regularly placed sodium/lithium carboxylate side chains instead of hydrocarbons. This block polyester shows significantly higher tensile strengths than its non-functionalized counterparts, with high elasticity and elastic recovery, providing beneficial properties for thermal reprocessing [125].

In an innovative contribution to the literature, Al Rashid and Koç address how additive manufacturing (AM) processes can synergistically impact and bring new life to discarded polymeric parts through distributed recycling and manufacturing. They reuse polymeric waste and turn it into valuable resources, generating low costs for specific AM and 3D printing applications for use in the aerospace, automotive, biomedical, sports, food, electronics, and construction sectors [126].

In their paper, Cherubini et al. aim to improve the process of obtaining elastomeric compounds by introducing used cooking oils, replacing typical mineral processing oils (lubricants). Cooking oils showed similar rheological behavior to traditional oils used in rubber production, resulting in the same processability of the resulting compound, as well as fracture and traction properties. These are promising results for applying circular economy principles by substituting mineral oils in manufacturing processing [127].

In their review, Doyle et al. analyze the critical considerations in designing circular products and the ecological engineering of polymeric foams. This includes the characterization of materials across a broader spectrum of molten polymer gas solutions, aging behavior, polymer chain adaptation, compression, and detailed modeling of the effects of cutting on cell nucleation, as well as the improvement of processing tools to achieve high results and defined pressure drop rates [128].

### 5.4. R-Strategies in the Lifecycle of Elastomers

R-strategies, or closed loops that indicate a material flow in a product’s lifecycle to an earlier node of the lifecycle, are generally understood to be circular economic processes [129]. Earlier frameworks for waste management outline the 3R framework, which stands for “Reduce, Reuse, Recycle”. Now, as many as 60 R-strategies can be found in the literature [130], and many effectively refer to similar processes. Cramer’s 10R framework (Refuse, Reduce, Renew, Reuse, Repair, Refurbish, Remanufacture, Repurpose, Recycle, Recover) [131], which is very close to Rieke’s et al. R-strategies [129], is differentiated enough to provide a more precise analysis than the 3R framework. We use this framework to evaluate the applicability of circular economy processes to the lifecycle of elastomer production. Table 3 defines each R-strategy and enumerates the elastomer-related literature in which applications related to these strategies are proposed. The literature cited here is incomplete but offers a general idea of which R-strategies are most commonly mentioned in the literature that ties elastomers to circular applications.

Circular elastomer production applications are focused on only a few of Cramer’s ten R-strategies [131]. Reduce and renew strategies are commonly cited in the literature. This is mainly because elastomers are often unusable after a certain period. Reusing, refurbishing, remanufacturing, or repurposing are rarely appropriate avenues. Redesigning the elastomers to limit the number of raw materials used or lengthen the product’s lifespan is more appropriate. Organizations employ research and development methods in light of these strategies. On the other hand, strategies that improve the recyclability of elastomers are also very commonly cited in the literature. Creating an elastomer that conserves value and can be easily recycled at the end of its lifespan is another objective that relies on research and development. What can be retained from the literature on circular strategies for elastomers is that the brunt of the effort in making an elastomer more circular is directed towards initiative-taking measures rather than reactive ones. While this is more interesting in terms of cost and energy saving eventually, it also requires firms to invest in research to make their products more circular early in the design process.

## 6. Conclusions and Perspectives

In this review the essential oils, d-limonene, β-pinene, terpenes, some menthol, β-citronellol, and terpenoids have the characteristics of being low in toxicity, inexpensive, and can be obtained by several extraction techniques; NR, PB and SBS modification with these compounds via *cross*-metathesis allows the synthesis of terpene-terminated oligomers and bio-based elastomers. They can be used in pharmaceutical, cosmetics, packaging, and chemical industries.

Of the mentioned methods, the synthesis of polyols by olefin metathesis reaction (depolymerization or degradation) has shown that both natural and industrial rubbers can be modified and degraded under mild conditions using natural resources with -OH groups and obtain bio-based diols and polyols (not derived from oil), which can be used for the synthesis of polymeric materials with elastomeric properties and the polyurethane industry.

The polyesters, polyethers, and their copolymers (segmented poly-ether-ester) arise as new sources with elastomer properties; these can be synthesized from bio-based resources such as castor oil, natural rubber, polylactic acid (PLA), lignocellulosic, limonene terpenes, fatty acids, vanillin, tetrahydrocannabinol, among others. These materials have the advantage of being biodegradable since the ester group is reported to be susceptible to microbial attacks. They have numerous applications, such as aerospace, construction, automobile, and, more recently, biomedical industries.

The current issues with elastomers in terms of sustainability are multifaceted and include resource dependency, energy-intensive production, end-of-life management, microplastic pollution, toxic additives, and a lack of circular economy practices. Circular elastomer production applications are focused on only a few of Cramer’s ten R-strategies. This is mainly because elastomers are often unusable after a certain period. Reusing, refurbishing, remanufacturing, or repurposing are rarely appropriate avenues. Creating an elastomer that conserves value and can be easily recycled at the end of its lifespan is another objective that relies on research and development; it also requires firms to invest in research to make their products more circular early in the design process.

Perspectives:

- We need standard norms that demand the use of bio-based elastomeric materials or the incorporation of recycled elastomer to produce new materials.
- Studies and techniques are needed to evaluate the formation and pollution of nano and microplastics released from elastomeric materials.
- Further research is required to explore R-strategies pathways, their classification, and the potential application of bio-based elastomers, such as “reuse–reduce–recycle”, to promote sustainable elastomer materials practices.
- Finally, for many elastomeric materials, sustainability in terms of environmental decomposition needs to be studied or reported; therefore, degradation and biodegradation studies are required. In addition to the fact that if a bio-based elastomer, this does not make it biodegradable or compostable, requiring studies for its classification.

## Figures and Tables

**Table 1:** Examples of terpene-terminated oligomers (bio-based elastomers) obtained by *cross*-metathesis reaction of NR and SBS with d-limonene and essential oils using Ru-carbene complexes.

Entry	Elastomer	CTA	Catalyst	[NR] ^b^ /[CTA]	Temp. (°C)	Yield ^c^ %	Molecular Weight		Ref.
							*M_n_* ^d^ (^1^H-NMR)	*M_n_* ^e^ (GPC)	
1	Natural rubber ^a^	*d*-limonene	Grubbs-2	1:1	50	80	722	779	[39]
2		Mandarin oil	Grubbs-1	1:1	100	74	16,836	17,554	
3		Mandarin oil	Grubbs-2	1:1	50	80	836	811	
4				5:1	50	95	3184	4745	
5				10:1	50	92	5675	7281	
6		Mandarin oil	I	1:1	80	95	9488	9800	[41]
7		Mandarin oil	II	1:1	80	95	10,941	10,398	[40]
8		Mandarin oil	III	1:1	80	96	10,693	10,700	[41]
9	Poly(styrene-*co*-butadiene) (SBS, 30% styrene)	*d*-limonene	I	1:1	80	96	289	295	
10		Orange	I	1:1	80	92	297	307	
11		Orange	Grubbs-1	1:1	50	93	285	325	[40]

^a^ *Hevea brasiliensis* natural rubber, Mn = 1.7 × 10^6^; molar ratio [C=C]/catalyst = 250, reaction time = 24 h. ^b^ Molar ratio of NR to CTA; chain transfer agent (essential oil). ^c^ Isolated yield of products. ^d^ Mn determined by ^1^H-NMR end groups analysis, where one unit of d-limonene is attached to the end-group of the isoprene oligomeric chain. ^e^ Number-average (Mn) molecular weights were calculated by gel permeation chromatography (GPC). The low molecular weights were calculated by SEC.

**Table 2:** Some examples of bio-based and biodegradable polyester and polyether elastomers and their outcome or application.

Bio-Based Source	Monomer Source	Monomer Obtained or Added	Synthesis Method	Poly Elastomer Obtained	Outcome/Application	Ref.
Castor plant	Castor oil ethoxylates	Mono-, di-, tri-, tetra-, and penta-esters consist of ricinoleate, oleate, linoleate, stearate, and combinations of such fatty acids and glycerol derivates.	Simple hydrolysis of monomers in milli-Q water and the separation was studied by two-dimensional liquid chromatography hyphenated with high-resolution mass spectrometry.	Di-, tri-, and tetra-esters	Polyether polyol initiators for the synthesis of polyurethanes with uses in coatings, adhesives, sealants, synthetic lubricants, and functional fluid	[88]
Plant-derived	Natural rubber from *Hevea brasiliensis*	Bicyclic β -Pinene (Polyterpene), acyclic *cis*-3-methyl pent-2-ene with diethyl α,β-unsaturated carbonyl compounds	*Cross*-metathesis reactions using fats and/or oils and inorganic catalysts	Unsaturated polyesters elastomers	Great potential as petrochemical alternatives include in perfume, flavor, and pharmaceutical industries	[89]
Hemicellulose from biomass	Bicyclic diol 1,2-O-isopropylidene-α-D-xylofuranose	D-xylose with ω-unsaturated fatty acids and alcohols	Acyclic diene metathesis polymerization	α,ω-unsaturated polyesters, and polyether’s	Promising bioplastic for sustainable packaging applications.	[90]
Plant-derived	4-hydroxy Benzaldehyde and vanillin with p-hydroxy acetophenone	Diphenol chalcone-derived monomers	Claisen–Schmidt condensation reactions with 1,6-dibromohexane	Co-polyether’s materials	Several applications in pharmaceutical compositions, microscopy, photosensitive materials, nonlinear optical materials, and crystal display liquid.	[91]
Renewable resource	Polylactic acid Hytrel commercial TPE	Glycidyl methacrylate, maleic anhydride	Melt processed reactions using ethylene butyl acrylate and ethylene methyl acrylate copolymers.	Thermoplastic Copolyester Elastomers	Substitution of plastics (i.e., Hytrel) into sustainable copolymers for commodity and engineering applications	[92]
Biomass	Lignocellulosic and limonene terpenes	1,6-hexanediol and 1,4-cyclohexanedimetha-nol	Self-condensation and polycondensation	Copolyether polyols	Ideal candidates for the preparation of a great variety of polyurethanes	[77]
Vegetal source	Biomass from corn	Various bio contents of poly propanediol and poly(tetramethylene ether) glycol as copolymer	Solvent-free process using tetramethyl xylylene diisocyanate	Biomass polyether diol-based polyurethane	Textile coatings	[93]
Bio-based organic chemistry	2,5-furan-dicarboxylic acid ethylene glycol	Poly(ethylene 2,5-furan dicarboxylate) and dimethyl furan-2,5-dicarboxylate	Via transesterification method with metal zinc powder as in-situ catalyst	Bio-Furan-based polyesters	Food and beverage packaging, clothing, and the car industry	[94]
Carbohydrates sources	Ethylene glycol and 2,5-furan-dicarboxylic acid	Poly(ethylene 2,5-furan-dicarboxylate) and poly(ethylene glycol)	Polytransesterification reaction	Series of copolyester/ethers furandicarboxylated	Packaging industry	[87]
Plant and vegetable origin.	Commercial greenpoxy 28 and woven jute fabric as reinforcement.	Allyl-functionalized ditertiary amine curing agent, a multifunctional thiol, and a radical photoinitiated	Epoxy/thiol-ene photopolymerization	Polyether–polythioether crosslinked co-network	Aerospace, nautical, automotive, packaging, and building industries	[95]

**Table 3:** According to Cramer and literature, R-Strategies tie circular concepts to elastomer lifecycles.

R-Strategy	Definition	Literature that Mentions R-Strategies and Ties Circular Concepts to Elastomer Lifecycles
Refuse	Prevent raw materials usage	--
Reduce	Decrease raw materials use	Kumawat et al. [106], Samir et al. [103], Utrera-Barrios et al. [101,105,120,121]
Renew	Redesign product was given circularity	Doyle et al. [128], Kumawat et al. [106], Samir et al. [103], Utrera-Barrios et al. [101,105,120], Zanchin and Leone [119]
Reuse	Use the product again (second-hand)	Hong and Chen [104]
Repair	Maintain and repair product	Utrera-Barrios et al. [101,105,120,121]
Refurbish	Revive product by replacing failed components	--
Remanufacture	Revive the product by replacing all components	Al Rashid and Koç [126]
Repurpose	Reuse product but with other function	--
Recycle	Salvage material streams with the highest possible value	Al Rashid and Koç [126], Cherubini et al. [127], Georgescu et al. [122], Gregory and Williams [125], Hong and Chen [104], Kouhi et al. [123], Kumawat et al. [106], Liu et al. [124], Zanchin and Leone [119]
Recover	Incinerate waste with energy recovery	--

## Data Availability

Not applicable.
